# Do Hippocampal Neurons Really Count for Comorbid Depression in Patients With Mesial Temporal Lobe Epilepsy and Hippocampal Sclerosis? A Histopathological Study

**DOI:** 10.3389/fnint.2021.747237

**Published:** 2021-11-30

**Authors:** Nathália Stela Visoná de Figueiredo, Anaclara Prada Jardim, Lenon Mazetto, Jeana Torres Corso Duarte, Sandra Mara Comper, Neide Barreira Alonso, Maria Helena da Silva Noffs, Carla Alessandra Scorza, Esper Abrão Cavalheiro, Ricardo Silva Centeno, Gerardo Maria de Araújo Filho, Elza Márcia Targas Yacubian

**Affiliations:** ^1^Department of Neurology and Neurosurgery, Universidade Federal de São Paulo (UNIFESP), São Paulo, Brazil; ^2^Department of Psychiatry and Medical Psychology, Faculdade de Medicina de São José do Rio Preto (FAMERP), São José do Rio Preto, Brazil

**Keywords:** histological evaluation, depression, mesial temporal epilepsy, hippocampal sclerosis (HS), neuronal count

## Abstract

Depression is the most frequent psychiatric comorbidity seen in mesial temporal lobe epilepsy (MTLE) patients with hippocampal sclerosis (HS). Moreover, the HS is the most frequent pathological hallmark in MTLE-HS. Although there is a well-documented hippocampal volumetric reduction in imaging studies of patients with major depressive disorder, in epilepsy with comorbid depression, the true role of the hippocampus is not entirely understood. This study aimed to verify if patients with unilateral MTLE-HS and the co-occurrence of depression have differences in neuronal density of the hippocampal sectors CA1–CA4. For this purpose, we used a histopathological approach. This was a pioneering study with patients having both clinical disorders. However, we found no difference in hippocampal neuronal density when depression co-occurs in patients with epilepsy. In this series, CA1 had the lowest counting in both groups, and HS ILAE Type 1 was the most prevalent. More studies using histological assessments are needed to clarify the physiopathology of depression in MTLE-HS.

## Introduction

Significant attention has been paid to comorbidities in epilepsy as they have important implications in the quality of life of patients. Psychiatric comorbidities are found in this context, and depression is the most prevalent (Hermann et al., 2000), occurring in approximately 22.9% of people with epilepsy (PWE) (Scott et al., 2017). Moreover, it can be clinically relevant in about 30–50% of patients with drug-resistant epilepsies throughout their lifetime (Gilliam and Kanner, 2002; Nogueira et al., 2017). This clinical situation occurs in mesial temporal lobe epilepsy (MTLE) with hippocampal sclerosis (HS), which frequently leads to surgical treatment (Blümcke et al., 2002). Given that depressive disorders affect between 2 and 21% of the world population and around 10.4% of the Brazilian population (Gutiérrez-Rojas et al., 2020), the higher frequency of depression in drug-resistant MTLE patients reinforces the importance of investigating the link between psychiatric diseases and epilepsy.

Although the hippocampus is classically associated with the processing of memory, it may be part of the pathological process of major depressive disorder as it is a highly plastic, as well as a stress-sensitive structure. In that sense, in this psychiatric disorder, it is expected a stress-induced response leading to an increase in glucocorticoid action, which interacts with neurotrophic factors and neurotransmitters (Campbell and Macqueen, 2004). In a final instance, these mechanisms could decrease hippocampal neurogenesis (Cameron and McKay, 1999) or stop the cellular cycle of the peripheral neurons of the hippocampus (Rogatsky et al., 1997). Besides the reversible remodeling of the hippocampus and a possible irreversible hippocampal cell death in major depressive disorder (Campbell and Macqueen, 2004), dendritic retraction and glial cell loss can contribute to the physiopathology of major depression (Czeh et al., 2001). In relation to the hippocampal sectors, moderate cellular apoptosis in CA1 and CA4 was reported in *post-mortem* studies of patients with this psychiatric disorder (Lucassen et al., 2001).

Therefore, MTLE-HS can be a neurobiological model for outlining the structures involved in the bidirectional relationship between epilepsy and depression (Kanner, 2011; Hesdorffer et al., 2012; Valente and Filho, 2013). However, there are few studies in the literature about the histopathological findings in patients having the co-occurrence of these medical conditions. There is a lack of knowledge about the expected histopathological profile in this clinical setting. For instance, it is not well-established if the hippocampal neuronal loss occurs more extensively in patients with comorbid depression than in those with only MTLE-HS and if any hippocampal sector is more affected than others. Moreover, the previous studies did not investigate the implication of the HS types, according to the new International League Against Epilepsy (ILAE) classification, for depression (Blümcke et al., 2013).

This study aimed to evaluate the aspects related to the histopathology of the hippocampus obtained after epilepsy surgery in a series of patients with MTLE-HS, comparing cases with vs. without comorbid depression. We analyzed these groups in relation to the neuronal densities of the hippocampus and its four sectors, according to the stratification of the Ammon’s horn, or *cornu Ammonis* (CA) in Latin. Moreover, we perform the histopathological classification of HS and the comparison of the groups based on the ILAE criteria (Blümcke et al., 2013). Finally, we compared the sectors of CA1–CA4 in each group to verify their internal pattern of neuronal density.

## Materials and Methods

This was an observational and retrospective case-control study. This series evaluated 58 adults with a previous diagnosis of MTLE with unilateral HS, according to the MRI analysis, who met the criteria for pharmacoresistant epilepsy (Kwan et al., 2010) and underwent surgery between 2005 and 2013. They did not have clinical or psychiatric illnesses that contraindicated the pre-surgical evaluations or this procedure. Patients without psychiatric comorbidities in the pre-surgical evaluation who presented signs and symptoms of depression or anxiety after the epilepsy surgery, a situation known as forced normalization (Sirven, 2016), were excluded. This was necessary to avoid bias in determining patients with comorbid depression, and future studies should further investigate this specific context. Patients with bilateral HS were also excluded. The Ethics Committee of the Federal University of São Paulo (UNIFESP) approved this study.

All patients underwent standardized pre-surgical evaluation in the epilepsy center, which included the following: video-electroencephalogram (VEEG) with sphenoidal electrodes; assessments by psychiatrists, neuropsychologists, and the quality-of-life team; MRI performed according to the local protocol for epilepsy. Epilepsy Surgery Inventory-55 (ESI-55) (Vickrey et al., 1992; Alonso et al., 2006) was applied for measuring the quality of life. Demographic data were also collected from the medical records of patients, based on clinical and neurological routine evaluations.

### Psychiatric Assessment

Psychiatrists with experience in epilepsy performed a semi-structured pre-surgical evaluation to diagnose the presence of comorbid depression. They were based on the Structured Clinical Interview for Diagnostic (SCID) (First et al., 1995; Del-Ben, 2001) of the Diagnostic and Statistical Manual of Mental Disorders (DSM) IV Axis-I (American Psychiatric Association, 2000), which was the standard assessment during the study period.

They also analyzed clinical characteristics related to interictal dysphoric disorder (Blumer et al., 2004), as a classical psychiatric perspective could not always identify comorbid depression in PWE. Their evaluation also analyzed the relationship of those symptoms with the occurrence of epileptic seizures, classifying them as ictal, perictal, or interictal.

After an extended formal and standardized psychiatric evaluation, the main groups were defined. Consequently, the inclusion criteria for the depression group were the presence of comorbid depression (with previous or current depressive signals and symptoms) based on the categorical diagnosis of the DSM-IV (American Psychiatric Association, 2000) and the occurrence of interictal dysphoric disorder if it was present (Blumer et al., 2004). Moreover, patients of this series were analyzed using a complete psychiatric assessment. As a result, patients with other mental conditions (e.g., anxiety, psychosis, personality disorder, sleep disorders, obsessive-compulsive disorder, schizophrenia, substance abuse disorder, and attention deficit hyperactivity disorder, among others) were also included in the study. Patients without pre-surgical depression who developed depressive symptoms, whether current or not, were excluded from the non-depression group to avoid biases.

Beck Depression Inventory (BDI) version I (Beck et al., 1961, 1988; Cunha, 2001) was also self-applied by the patients. Psychiatric data collected from the medical records of patients included the following: familiar history of psychiatric illnesses; ideation(s) and suicide attempt(s); occurrence of psychogenic non-epileptic seizures (PNES); type of psychiatric medications by psychotropic category (antidepressants, antipsychotics, anxiolytics, or none) and by active substance, both used throughout life and during the VEEG.

### Histopathological Assessment

The standard recommendation by the ILAE to define the HS types (Blümcke et al., 2013) in the histopathological analysis was followed. The entire hippocampus resected *en bloc* by neurosurgery was dissected in coronal sections of 5 mm, using the middle portion of the hippocampus body in its coronal axis as an anatomical indicator. Then, the tissue was fixed in 10% formalin for 24 h and routinely processed in liquid paraffin. The median portion of the hippocampal body was then cut into 5 μm thick sections, which were submitted to immunohistochemistry to detect neurons using an anti-NeuN antibody (Chemicon Temecula, United States, dilution 1:1000) and hematoxylin counterstaining. Neuronal densities were determined by counting the cell bodies of all NeuN-stained neurons, despite the variation in morphology and size. The evaluators were independent and unaware of the clinical cases analyzed; they applied the Image J software (developed by Wayne Rasband, National Institutes of Health, United States^1^) to perform the quantitative and manual analysis of the neurons on the computer screen. For this, four areas were randomly chosen in the sectors CA1, CA2, CA3, and CA4 at 20x to enable visual counting in each hippocampal sector. The neuronal density of each sector was calculated, and whole hippocampus density was defined as a mean of the four sectors. The HS type was qualitatively determined according to the ILAE classification (Blümcke et al., 2013). We also used in the comparison of the main groups, a control group of 12 hippocampal tissues obtained after *post-mortem* time of 5.4 ± 3.4 h from six men and six women (mean age of 58.7 ± 15.43 years), who have had severe acute clinical conditions leading to death (e.g., heart attack, acute lung edema, aortic dissection, hemorrhagic shock, etc.). They did not have any presumed neurological (as epilepsy) or psychiatric diseases, including depression. However, there was a lack of structured psychiatric evaluation before their death to confirm the precisely mental status of each of them.

### Statistical Analysis

We used Fisher’s exact test for categorical variables and the Mann-Whitney test for non-parametric numerical variables to analyze clinical and demographic data. To compare the histopathological findings of the depression and non-depression groups, we used Fisher’s exact test to compare HS subtypes and the Mann-Whitney test to analyze the neuronal densities of the hippocampus and its four sectors. All data from the histopathological analysis were converted to *Z*-scores (Zs), which were calculated using the average and *SD* of the control group.

Furthermore, we compared the neuronal densities of the two groups on the four hippocampal sectors, using Friedman’s test. Here, we used the Zs, which allowed the investigation of any different histopathological findings concerning the pattern expected for the MTLE-HS. Finally, we compared HS ILAE Type 1 vs. the combination of atypical HS types plus no-HS using the test of proportions.

## Results

### Neuro-Psychiatric Results

Fifty-eight patients with MTLE-HS were included, being 20 (34.5%) with comorbid depression. All clinical, neuropsychiatric, and quality-of-life data of this series are shown in Tables 1, 2.

**TABLE 1:** Clinical and neurological profile of this series of patients with mesial temporal lobe epilepsy and hippocampal sclerosis, with and without comorbid depression.

	Depression group	Non-depression group	Statistics
Total cases	20 (34.5%)	38 (65.5%)	*N* = 58
Woman (N/%)	13 (65.0%)	16 (42.1%)	*p* = 0.167^b^
HS-side (N/%)			*p* = 0.578^b^
Right	10 (50.0%)	23 (60.5%)	
Left	10 (50.0%)	15 (39.5%)	
Surgery type (N/%)			*p* = 0.450^b^
Right CAH	10 (50.0%)	23 (60.5%)	
Left CAH	7 (35.0%)	13 (34.2%)	
Epilepsy onset age (mean ± SD)	14.3 ± 12.79	15.0 ± 8.07	*p* = 0.447^b^
Age at surgery (mean ± SD)	41.2 ± 9.58	35.0 ± 11.80	*p* = 0.039^b^
Epilepsy duration (mean ± SD)	26.9 ± 12.28	20.0 ± 11.98	*p* = 0.036^b^
**Seizure frequency**			
FIAS per month (mean ± SD)	5.3 ± 4.81	4.3 ± 4.77	*p* = 0.441^b^
Focal evolving to bilateral tonic-clonic (>20/lifetime) (%)	65.0%	47.3%	*p* = 0.349^a^
**IPI (%)**			
Total	55.0%	63.3%	*p* = 0.583^a^
Febrile seizures	37.5%	36.4%	*p* = 0.685^a^
Afebrile seizures	37.5%	18.2%	*p* = 0.685^a^
ASM (mean ± SD)	2.0 ± 0.69	2.1 ± 0.73	*p* = 0.486^b^

*ASM, antiseizure medication; CAH, corticoamigdalohyppocampectomy; FIAS, focal impaired aware seizures; HS, hippocampal sclerosis; ILAE, International League Against Epilepsy; IPI, initial precipitating injury; N, absolute number; p, p-value; SD, standard deviation.*

*^a^Fisher’s exact test.*

*^b^Mann-Whitney test.*

**TABLE 2:** Psychiatric and quality-of-life profile of this series of patients with mesial temporal lobe epilepsy and hippocampal sclerosis, with and without comorbid depression.

	Depression group	Non-depression group	Statistics
Total cases	20 (34.5%)	38 (65.5%)	*N* = 58
**ESI-55 (mean ± SD)**			
Overall quality of life	54.1 ± 16.82	64.2 ± 15.80	*p* = 0.030^1^
Emotional well-being	43.1 ± 24.76	66.7 ± 17.43	*p* = 0.001^1^
Energy/fatigue	48.6 ± 24.61	67.6 ± 14.32	*p* = 0.002^1^
Role limitations (physical problems)	40.0 ± 40.55	69.7 ± 35.40	*p* = 0.014^1^
BDI-I (mean ± SD)	16.5 ± 10.04	8.8 ± 8.26	*p* = 0.002^1^
>16 points (N/%)	9 (45.0%)	5 (13.2%)	*p* = 0.011^2^
Pre-surgical anxiety (N/%)	9 (45.0%)	0 (0.0%)	*p* < 0.001^2^
**Psychiatric history (N/%)**			
Familiar	3 (15.0%)	1 (2.7%)	*p* = 0.114^2^
Personal	19 (95.0%)	8 (21.1%)	*p* < 0.001^2^
Psy. comorbidities (N/%)			*p* = 0.002^2^
Total	7 (35.0%)	1 (2.7%)	
Personality disorders	4 (20.0%)	0 (0.0%)	
OCD	3 (15.0%)	0 (0.0%)	
Psychosis	1 (5.0%)	1 (2.7%)	
**Suicide (N/%)**			
Ideation	6 (30.0%)	0 (0.0%)	*p* = 0.001^2^
Attempts	5 (25.0%)	0 (0.0%)	*p* = 0.004^2^
PNES before surgery (N/%)	5 (35.0%)	0 (0.0%)	*p* = 0.004^2^
Psy. medications (%)	60.0%^a^/35.0%^b^	2.6%^a^/0.0%^b^	*p* < 0.001^a1^/*p* < 0.001^b1^
**Antidepressants (%)**			
sertraline	15.0%^a^/10.0%^b^	0.0%^a^/0.0%^b^	*p* = 0.034^a1^/*p* = NM^b^
fluoxetine	45.0%^a^/15.0%^b^	0.0%^a^/0.0%^b^	*p* < 0.001^a1^/*p* = NM^b^
Antipsychotics (%)	10.0%^a^/5.0%^b^	5.3%^a^/0.0%^b^	*p* = 0.602^a1^/*p* = 0.345^b1^
Anxiolytics (%)	25.0%^a^/15.0%^b^	7.9%^a^/5.3%^b^	*p* = 0.110^a1^/*p* = 0.328^b1^
None (%)	35.0%^a^/55.0%^b^	86.8%^a^/94.7%^b^	*p* < 0.001^a1^/*p* = 0.001^b1^

*BDI, Beck Depression Inventory; ESI-55, Epilepsy Surgery Inventory-55; HS, hippocampal sclerosis; ILAE, International League Against Epilepsy; N, absolute number; NM, non-measured; OCD, Obsessive-compulsive disorders; p, p-value; PNES, psychogenic non-epileptic seizures; psy, psychiatric; SD, standard deviation; VEEG, video-electroencephalogram.*

*^a^Throughout lifetime.*

*^b^At VEEG.*

*^1^Mann-Whitney test.*

*^2^Fisher’s exact test.*

### Histopathological Results

Hippocampal neuron density (in ×10^–4^/μm^2)^ was similar between the groups, being 0.94 ± 0.44 (Zs = −3.53 ± 0.8) in depression group and 1.01 ± 0.56 (Zs = −3.39 ± 1.01) in non-depression group (*p* = 0.798; Table 3). Regarding the hippocampal sectors, the lowest neuronal counting was found in CA1 for both groups (Zs = −4.59 vs. Zs = −4.44, respectively; *p* = 0.718; showed in Table 3).

**TABLE 3:** Neuronal densities of the entire hippocampus, as well as its four sectors, in absolute numbers and *Z*-scores, comparing depression and non-depression groups.

	Neuronal density (absolute number)^a^			Neuronal density (Z-scores)		
	Non-depression group^b^	Depression group^b^	*p*-value^1^	Non-depression group^b^	Depression group^b^	*p*-value^1^
CA1	0.72 ± 0.69	0.65 ± 0.37	0.718	−4.44 ± 1.52	−4.59 ± 0.829	0.718
CA2	1.45 ± 0.79	1.34 ± 0.85	0.508	−2.78 ± 1.17	−2.93 ± 1.26	0.508
CA3	1.31 ± 0.85	1.06 ± 0.62	0.356	−2.60 ± 1.32	−2.97 ± 0.95	0.456
CA4	0.67 ± 0.46	0.73 ± 0.51	0.823	−2.93 ± 0.94	−2.79 ± 1.04	0.823
Total*	1.01 ± 0.56	0.94 ± 0.44	0.798	−3.39 ± 1.01	−3.53 ± 0.80	0.798

*CA, “Ammons” horn’ in Latin (cornu Ammonis); CA1, the hippocampal sector 1; CA2, the hippocampal sector 2; CA3, the hippocampal sector 3; CA4, the hippocampal sector 4; NM, non-measured.*

*^a^Absolute number expressed as ×10^–4^/μm^2^.*

*^b^M ± SD.*

*^1^Mann-Whitney test.*

*Referred to the sum of CA1, CA2, CA3, and CA4.

**FIGURE 1:**
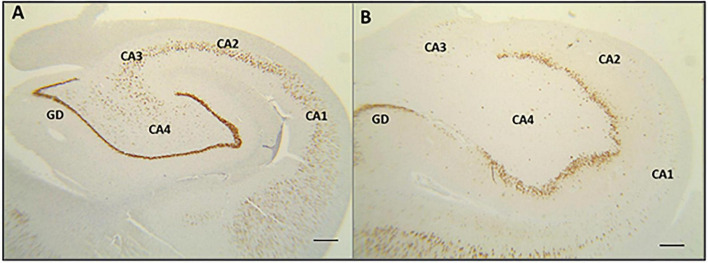
Image of hippocampal tissue comparing specimens from healthy control and a patient with temporal lobe epilepsy associated with hippocampal sclerosis (MTLE-HS). **(A)** Normal hippocampal neuronal density, as well as sectors delimitations (from CA1 to CA4), obtained from a healthy individual (NeuN staining and 2x objective magnification). **(B)** Reduction of the hippocampal neuronal density in a patient with MTLE-HS. There was no histopathological difference in neuronal densities between MTLE-HS patients with and without comorbid depression.

Hippocampal sclerosis ILAE Type 1 was the most prevalent in depression and non-depression groups (75 and 86.9%, respectively; *p* = 0.311). On the other hand, HS ILAE Type 2 was four times higher in the depression group (20 vs. 5.3%). Only one patient from the non-depression group has the hippocampus tissue classified as HS ILAE Type 3 (Table 4). Although all MRIs from our patients had clear signals of HS (hippocampal atrophy with a higher signal in T2, as well as a FLAIR signal, and the involvement of other structures nearby the hippocampus itself) diagnosed by a neuroradiologist with experience in epilepsy, three of them had no significant neuron loss and were classified as no-HS. Figure 1 illustrates the hippocampal tissue specimens from a control and an MTLE-HS patient analyzed in this study. The depression group had lower Zs than non-depression only in the CA1 subfield (for all results *p* < 0.05; Table 5). Moreover, HS ILAE Type 1 was the predominant compared with other types for both groups (*p* = 0.004 and *p* < 0.001, respectively; Table 6).

**TABLE 4:** Histopathological classification of the patients evaluated in this series, according to the International League Against Epilepsy (ILAE) criteria for the definition of the hippocampal sclerosis type.

HS ILAE Type (N/%)	Non-depression group	Depression group	*p*-value^a^
HS ILAE Type 1	33 (86.9)	15 (75.0)	0.311
HS ILAE Type 2	2 (5.3)	4 (20.0)	
HS ILAE Type 3	1 (2.6)	0 (0.0)	
No-HS	2 (5.3)	1 (5.0)	

*HS, hippocampal sclerosis; ILAE, International League Against Epilepsy.*

*^a^Fisher’s exact test.*

**TABLE 5:** Neuronal densities of the four hippocampal sectors, in *Z*-scores, comparing each main group (depression and non-depression) internally.

	Neuronal density (Z-scores)			
	NDG^a^	*p*-value^1^	DG^a^	*p*-value^1^
CA1	−4.44 ± 1.52	<0.001	−4.59 ± 0.829	< 0.001
CA2	−2.78 ± 1.17		−2.93 ± 1.26	
CA3	−2.60 ± 1.32		−2.97 ± 0.95	
CA4	−2.93 ± 0.94		−2.79 ± 1.04	
*Notes*	CA1≠CA2 (*p* < 0.001)		CA1≠CA2 (*p* < 0.001)	
	CA1≠CA3 (*p* < 0.001)		CA1≠CA3 (*p* = 0.002)	
	CA1≠CA4 (*p* < 0.001)		CA1≠CA4 (*p* < 0.001)	
	CA2 = CA3 (*p* = 1.000)		CA2 = CA3 (*p* = 0.332)	
	CA2 = CA4 (*p* = 0.457)		CA2 = CA4 (*p* = 0.503)	
	CA3 = CA4 (*p* = 1.000)		CA3 = CA4 (*p* = 0.332)	

*CA, “Ammons” horn’ in Latin (cornu Ammonis); CA1, the hippocampal sector 1; CA2, the hippocampal sector 2; CA3, the hippocampal sector 3; CA4, the hippocampal sector 4; DG, depression group; NDG, non-depression group.*

*^a^M ± SD.*

*^1^Friedman’s test.*

*In the notes, we showed the pairwise comparisons between the four hippocampal sectors.*

**TABLE 6:** Histopathological classification of the patients evaluated in this study, according to the International League Against Epilepsy (ILAE) criteria for the definition of the hippocampal sclerosis type – the comparison between type 1 versus others.

HS ILAE Type (N/%)	Non-depression group	*p*-value^a^	Depression group	*p*-value^a^
HS ILAE Type 1	33 (86.9)	<0.001	15 (75.0)	0.004
Others*	5 (13.2)		5 (25.0)	

*HS, hippocampal sclerosis; ILAE, International League Against Epilepsy.*

*^a^Test of proportion.*

*Referred to the sum of HS ILAE Type 2, HS ILAE Type 3, and no-HS.

## Discussion

### Analysis of the Neuropsychiatric Findings

In this series, comorbid depression was found in 34.5% of patients with MTLE, who presented exclusively unilateral HS as the structural substrate. This finding agrees with literature, in which depression varies from 21.6 to 39.6% (Sanchez-Gistau et al., 2012; Koch-Stoecker et al., 2017; Nogueira et al., 2017). Those series were more heterogeneous, having TLE patients with different etiologies. Moreover, a strength of our study is a DSM-IV-based diagnosis of depression by psychiatrists, considered the gold standard at the time of the clinical evaluation (American Psychiatric Association, 2000).

Patients with comorbid depression of this series were older at the time of surgery and had a longer duration of their epilepsy, which was previously reported by our group (de Araújo Filho et al., 2011, 2012). Epilepsy surgery in patients with depression is often performed later than in patients without this comorbidity (Briellmann et al., 2007; Sanchez-Gistau et al., 2012). However, the literature does not refer to formal contraindication for surgery in those patients (Kanner et al., 2009, 2010). Thus, these patients should not be neglected regarding the offer of additional surgical treatments. It also reports the need to include mental care before, during, and after epilepsy surgery to achieve a better post-surgical outcome.

All patients from the depression group used antidepressant medications, both throughout life and during the pre-surgical evaluation. This shows adequate mental care provided to those patients in our epilepsy service by the psychiatrists. Then, this series differs from other studies, which pointed out an insufficiency of this type of treatment being offered to PWE (Sanchez-Gistau et al., 2012; Nogueira et al., 2017). In this study, patients in the depression group received similar ASMs treatment to the non-depression group. However, the use of ASMs with positive psychotropic properties could have somehow affected the histopathological findings, at least influencing a possible better evolution of the comorbid depression in those patients.

As was expected, the depression group had higher scores in the BDI than the non-depression group, using a new proposed cut-off of > 16 points (Gill et al., 2017), which was more specific for PWE. Moreover, the BDI shows that, in this series, the presence of current depressive symptoms was also found, which complete the psychiatric profile of those patients, as it is a qualitative assessment used in addition to the categorical criteria of DSM (American Psychiatric Association, 2000) for diagnosis. Furthermore, the quality-of-life assessment showed lower scores in the depression group in general quality-of-life and emotional well-being, in agreement with a previous study (de Araújo Filho et al., 2011). ESI-55 application confirmed the expected profile in the clinical setting of MTLE-HS with comorbid depression and validated our series as an accurate sample of the population we aimed to research.

Finally, patients with depression also had other psychiatric comorbidities, especially anxiety, which is cited in previous studies (Koch-Stoecker et al., 2017; Nogueira et al., 2017) as frequent comorbidity in TLE patients. In opposition to the literature (Nogueira et al., 2017), familiar history of psychiatric illness was not significant in this study, possibly due to a memory bias of patients and their families.

### Analysis of the Histopathological Findings

This series showed hippocampal neuronal density reduced in patients with MTLE-HS and independently of the presence of depression as psychiatric comorbidity. Although this study found a negative result related to the histopathological analysis, there are fewer similar studies in the literature, showing that there is still a lack of knowledge in this field. Our data on the histological similarity between cases with and without depression confirms the findings of a previous report by Kandratavicius et al. (2014). We saw a reduction in all hippocampal sectors, especially in CA1, when we compared cases with and without comorbidity. Moreover, HS ILAE Type 1 was the principal finding in the qualitative analysis for both groups.

Autopsy studies of patients with major depressive disorder, who committed suicide, did not show hippocampal cell reduction or loss (Stockmeier et al., 2004; Cobb et al., 2013). The study of Cobb et al. (2013) postulated that the hippocampal volumetric reduction, which occurs in both recurrent and chronic stages of this psychiatric disorder, was related to the duration of the disease and not to a reduction in the number of total neurons (Cobb et al., 2013). For them, neither the hippocampus nor its four sectors had any different cell density in patients with major depressive disorder and healthy subjects. Then, Cobb et al. (2013) denied an initially formulated hypothesis of a pathological cellular basis for the reduced hippocampus volume (Cobb et al., 2013). In a final instance, this group of authors considered that the hippocampal volumetric reduction in major depressive disorder is a consequence of a reduction in the neuropil (white matter) and not in the hippocampal cells (neurons and glial cells) *per se* (Cobb et al., 2013). Following previous literature data and trying to find a common denominator between the psychiatric and neurological perspectives, this study suggests that the hippocampal volumetric reduction in MTLE-HS is part of the histopathological process of epilepsy *per se* and is not probably related to the comorbid depression in this clinical context.

Finally, patients in the depression group were also treated with antidepressant medications with a mechanism of action on the serotonin-modulating monoaminergic pathways. They may be responsible for a neuroprotective and, ultimately, a “neuronal sparing” factor in patients with depression, as has been already suggested by *in vitro* studies with human hippocampal tissue (Campbell and Macqueen, 2004; Ferrari and Villa, 2017). Thus, the use of psychiatric medications in this series could have had some implication on the neuronal density of the depression group, which was statistically equal to the non-depression group.

### Limitations

A group of patients who had major depressive disorder without epilepsy was not included in this series to be compared with MTLE-HS with and without depression. For further analysis, hippocampal specimens obtained from autopsies of patients who had been diagnosed with major depressive disorder in life would enrich the histological assessment.

 The tissue analysis performed in this study aimed to count hippocampal neurons, but other cells that may have an additional role in this context were not evaluated. Therefore, it would be essential to analyze whether the reduction in hippocampal volume found in MTLE-HS patients could have a different profile related to the glia in the presence or absence of comorbid depression. In this sense, some authors have already speculated about the more prominent participation of chaperones related to glial cells in this setting of depression comorbid to MTLE-HS (Kandratavicius et al., 2014, 2015).

Once the analysis was restricted to the hippocampus and its four sectors, the evaluation of other structures potentially involved in the physiopathology of depression co-occurring with MTLE-HS are also missing. Future studies should analyze the dentate gyrus, which is associated with neurogenesis and should be a region where antidepressant treatment can achieve a “neuroprotective effect” (Campbell and Macqueen, 2004; Ferrari and Villa, 2017). The amygdala should also be evaluated, although its histological analysis is technically more challenging.

The methodology of this study also brought some limitations related to information bias since some data were collected retrospectively from medical records, and some were informed by patients/relatives, as well as it had a small number of patients included. Therefore, prospective studies should follow the patients to achieve higher accuracy and need to include more individuals. After all, multiple comparisons were made in this study and needed to be carefully interpreted in a setting of positive results, even if the main result found here was a negative one.

### New Contributions to the Literature

In this series of MTLE patients with exclusively unilateral HS, the prevalence of comorbid depression was similar to previous findings in other series worldwide. However, this study found no difference between the depression and non-depression groups regarding the hippocampal neuronal densities. In addition, there was no difference in the histopathological classification of HS according to the ILAE criteria (2013), with HS ILAE Type 1 prevailing sclerosis in both groups. The evaluation of hippocampal sectors within each group showed a lower neuronal density in CA1 of both. More contributions are needed regarding the analysis of hippocampal tissue when depression is present as a comorbidity in patients with epilepsy. These frontiers between neurology and psychiatry still deserve further investigation.

## Data Availability Statement

The original contributions presented in the study are included in the article/supplementary material, further inquiries can be directed to the corresponding author/s.

## Ethics Statement

The studies involving human participants were reviewed and approved by the Universidade Federal de São Paulo. Written informed consent for participation was not required for this study in accordance with the national legislation and the institutional requirements.

## Author Contributions

NV, AJ, and EY: conception and study design. NV: statistical analysis. NV and EY: interpretation of the results and drafting the manuscript work or revising it critically for important content. All authors: data collection, acquisition and approval of final version to be published and agreement to be accountable for the integrity and accuracy of all aspects of the work.

## Conflict of Interest

The authors declare that the research was conducted in the absence of any commercial or financial relationships that could be construed as a potential conflict of interest.

## Publisher’s Note

All claims expressed in this article are solely those of the authors and do not necessarily represent those of their affiliated organizations, or those of the publisher, the editors and the reviewers. Any product that may be evaluated in this article, or claim that may be made by its manufacturer, is not guaranteed or endorsed by the publisher.

## Footnotes

1
https://imagej.nih.gov/ij/index.html
